# Neural correlates of Sudoku play: a systematic review of brain imaging studies

**DOI:** 10.3389/fnimg.2026.1756394

**Published:** 2026-04-20

**Authors:** Morgan J. Williams, Ellie J. Williamson, Samantha Jane Brooks

**Affiliations:** 1 Departments of Psychology, University of Leicester, Leicester, United Kingdom; 2 School of Psychology, Liverpool John Moores University, Liverpool, United Kingdom; 3 NeuRL Department of Psychology, University of Witwatersrand, Johannesburg, South Africa

**Keywords:** cognitive training, neuroimaging, problem solving, Sudoku, working memory

## Abstract

**Introduction:**

Sudoku is a popular logic-based puzzle that requires sustained attention, working memory, and rule-based reasoning. Despite its widespread use, the neural processes supporting Sudoku play have not been systematically synthesised, limiting understanding of its potential applications beyond leisure.

**Methods:**

This systematic review aimed to examine the neural correlates of Sudoku solving and to evaluate its potential relevance as a cognitive training paradigm. Six neuroimaging studies were included (five fMRI, and one fNIRS).

**Results:**

Across haemodynamic studies, Sudoku solving consistently engaged frontoparietal networks, including the dorsolateral prefrontal cortex (DLPFC) and parietal regions implicated in executive control and visuospatial working memory, alongside activation of the anterior cingulate cortex (ACC), associated with performance monitoring and cognitive control.

**Discussion:**

The included fNIRS study provided converging evidence of increased prefrontal activation during Sudoku solving under more ecologically valid conditions. Together, these findings suggest that Sudoku play recruits distributed neural systems supporting cognitive control, monitoring, and memory processes. While the limited number and heterogeneity of studies preclude firm conclusions regarding efficacy, the observed neural engagement highlights Sudoku as a candidate task for probing executive function and self-regulatory processes in both healthy and clinical populations.

## 1 Introduction

Puzzles and other cognitive activities are widely recognised as effective forms of mental stimulation that engage various aspects of thinking, memory and problem-solving. Engaging in these activities has been shown to improve or maintain working memory, attention, verbal fluency and executive function across different age groups (Cegolon and Jenkins, 2022; Ferreira et al., 2015; Baek et al., 2008; Hambrick et al., 1999; Iizuka et al., 2019; Lin et al., 2023; Pillai et al., 2011). One of these cognitive puzzles is sudoku, a game popularised in the 1980s by Japanese company Nikoli, based on the concept of Latin squares, introduced by Swiss mathematician Leonhard Euler in the 18th Century. It was first published in 1979 as a puzzle called Number Place by Howard Garns in Dell Magazines, and later the president of Nikoli, Maki Kaji, contributed to the popularisation of the game under the name Sūji wa dokushin ni kagiru, meaning “the numbers must be single”, later shortened to Sudoku. The game is now considered one of the most popular puzzles around (Rosenhouse and Taalman, 2012) and is a well-known household term.

**Figure 1:**
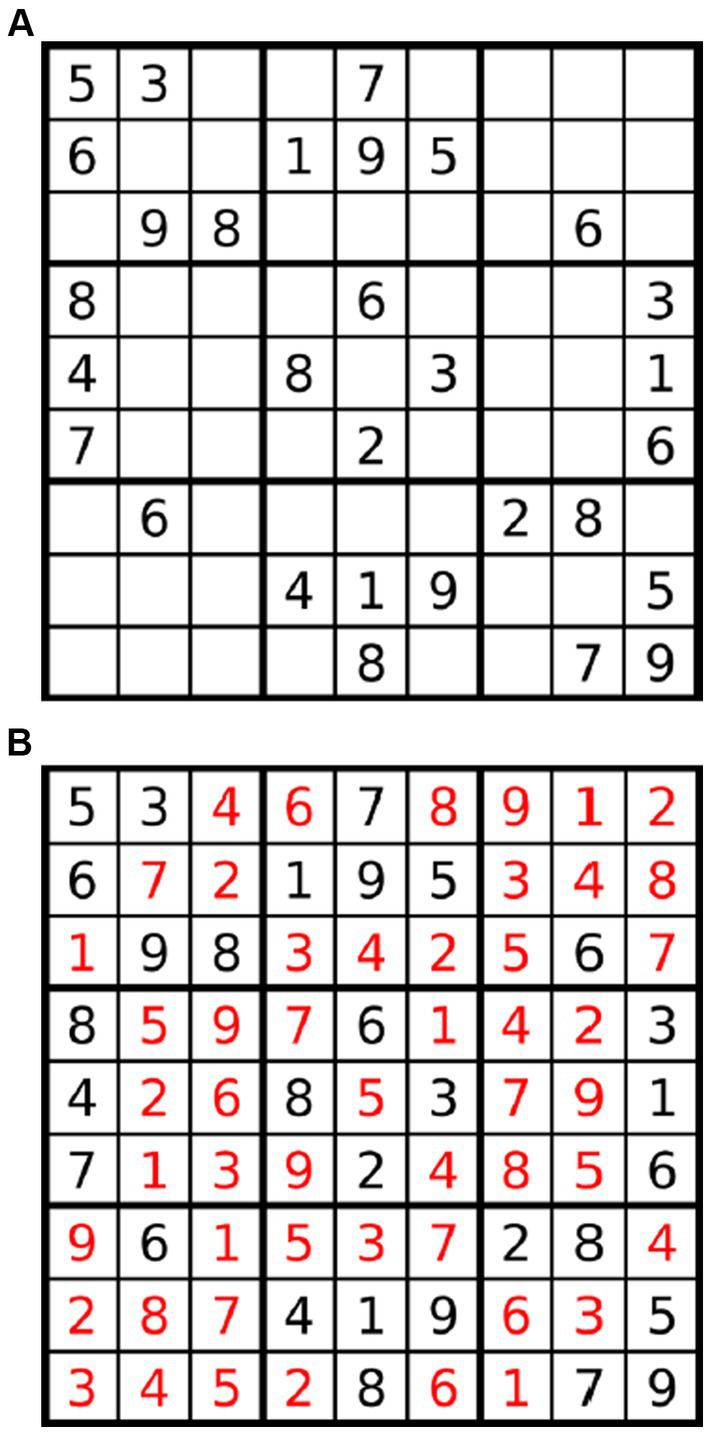
Example Sudoku puzzle (adapted from Wikipedia, 2023, under CC BY-SA 3.0. license). **(A)** A typical Sudoku puzzle: **(B)** The solution to the puzzle above.

Sudoku is a logic-based puzzle game played on a 9 × 9 grid, subdivided into nine 3 × 3 boxes (Figure 1). The objective is to fill the grid so that each row, column and box contains the digits 1 through 9 exactly once, starting from a partially filled grid. Sudoku can be solved using various techniques, including logical deduction, backtracking, constraint propagation, pattern recognition and advanced algorithms such as evolutionary computation and graph colouring (Felgenhauer and Jarvis, 2006; Mantere and Koljonen, 2007; Pal et al., 2018; Soto et al., 2015; Li, 2025).

Solving Sudoku requires the integration of several core cognitive functions. Previous literature has highlighted a significant relationship between Sudoku performance and working memory (WM) capacity in both younger and older adults, suggesting that Sudoku relies heavily on WM processes (Chang and Gibson, 2011; Grabbe, 2011; Grabbe, 2017). Sudoku requires a balance between storing spatial and numerical information and actively manipulating it during problem solving. As puzzle difficulty increases, the demand on WM for both storage and processing also increases, highlighting the importance of efficient strategy use (Leu et al., 2014). In addition to WM, Sudoku engages multiple executive functions, including planning and sequencing actions, cognitive flexibility to shift strategies, and the maintenance of potential solutions while adhering to rule constraints (Ashlesh et al., 2020; Grabbe, 2011; Kalia et al., 2019).

Sudoku is fundamentally a logic-based puzzle that relies on logical reasoning, defined as the process of drawing valid conclusions from a set of rules or constraints. Within this framework, deductive reasoning plays a central role, as solvers systematically eliminate possibilities and infer correct placements based on the current state of the grid. In addition to deduction, more complex solving strategies may involve hypothetical reasoning, whereby individuals temporarily test potential solutions and evaluate their consequences before committing to a response (Chi and Lange, 2012; Maji and Pal, 2014; Wagatsuma, 2013). These processes require multi-step inference and the ability to anticipate how each decision constrains future possibilities, placing substantial demands on cognitive resources.

Research using Sudoku as a cognitive task provides evidence that engagement with the puzzle is associated with performance in domains such as working memory, reasoning, and executive functions. Cross-sectional analyses have shown that regular engagement with Sudoku and similar puzzle activities is associated with improved verbal fluency, numeracy and memory (Litwin et al., 2017). Experimental work further demonstrates that Sudoku performance correlates with working memory capacity (Grabbe, 2011), and task manipulations confirm its reliance on executive functions. Intervention studies provide mixed but encouraging evidence, although methodological limitations often restrict conclusions regarding transfer effects (Corbett et al., 2024; Rodas et al., 2024). While these behavioural findings suggest potential cognitive benefits, they provide limited insight into the neural mechanisms that support Sudoku performance.

Neuroimaging modalities offer critical insight into how these cognitive processes are instantiated in the brain. Functional magnetic resonance imaging (fMRI) and functional near-infrared spectroscopy (fNIRS) studies indicate that Sudoku solving engages distributed frontoparietal networks, including the dorsolateral prefrontal cortex, anterior cingulate cortex, and posterior parietal regions, which are consistently implicated in working memory and executive function tasks (Nombela et al., 2011; Qiu et al., 2018). These findings suggest that Sudoku recruits coordinated neural systems supporting cognitive control, monitoring, and goal-directed behaviour.

Despite the growing body of research on Sudoku and cognition, the neural substrates supporting Sudoku performance remain dispersed across studies, modalities, and populations, making it difficult to form a cohesive understanding of the underlying mechanisms. A systematic synthesis is therefore needed to map the distributed neural systems, particularly frontoparietal and executive networks, that support Sudoku solving. Accordingly, this systematic review aims to identify and synthesise neuroimaging studies that have examined Sudoku play in order to characterise its neural correlates.

## 2 Methodology

This systematic review was conducted and reported in accordance with the Preferred Reporting Items for Systematic Reviews and Meta-Analyses (PRISMA) guidelines. PubMed was used to systematically search for publications. The search strategy included the terms “Sudoku” AND “fMRI” OR “EEG” OR “MEG” OR “SPECT” OR “fNIRS.” The search was limited to peer-revived articles published in English that made use of neuroimaging technologies and involved a Sudoku or Sudoku-like puzzles as part of the experimental task. Reference lists of publications were examined, and the search was not restricted to any specific study design.

Exclusion criteria were also applied. Studies that used physiological measures such as skin conductance and heart rate measurements were excluded, to focus on brain imaging measures only. Additionally, studies using other puzzles or games (such as chess; crosswords; generic brain games) were excluded.

**Figure 2:**
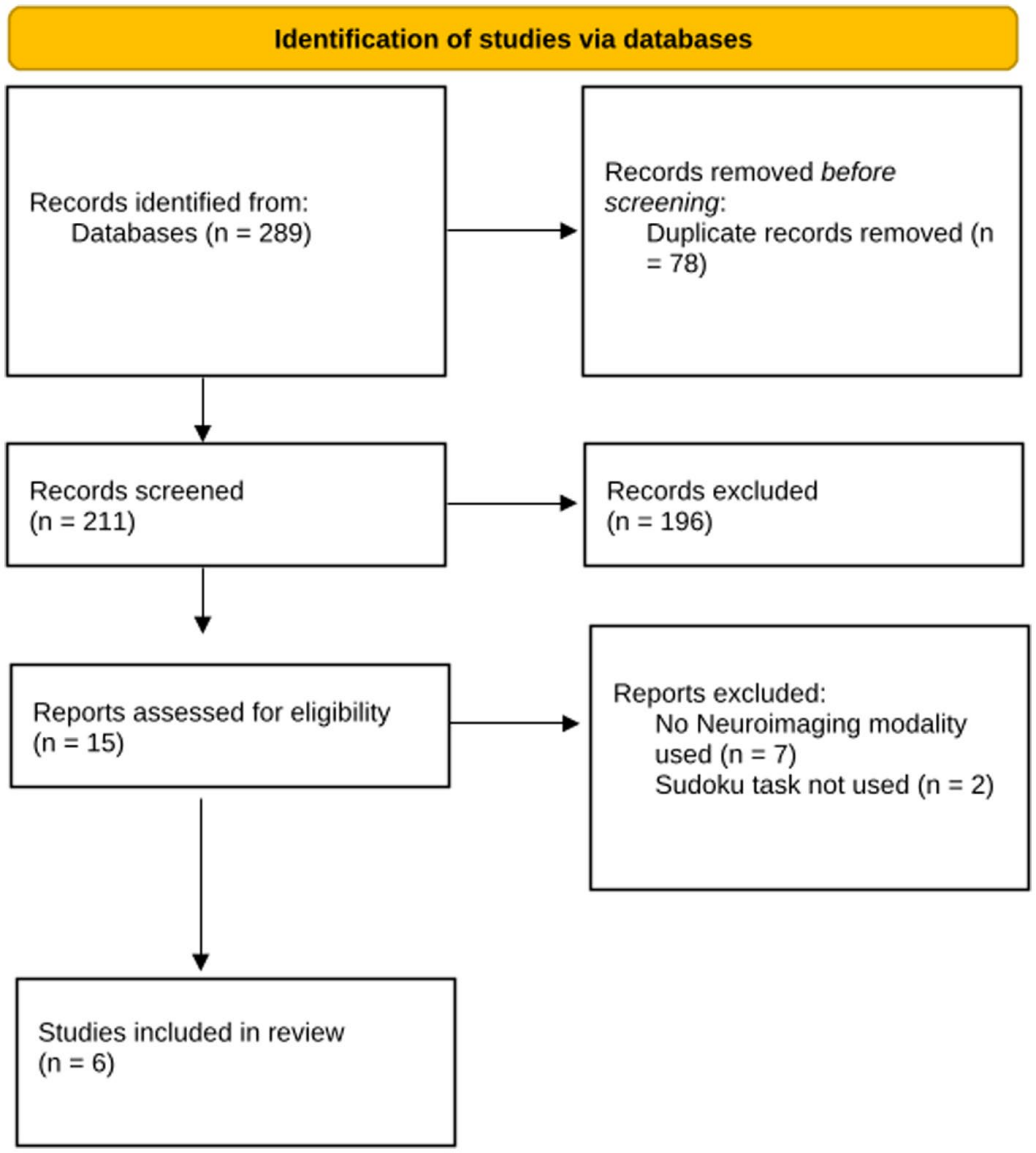
PRISMA study inclusion flow diagram.

The search of any studies with no date limitation was executed on 9th July 2025 and data extraction was completed on 13th August 2025. In total the searches yielded 289 results. Duplicates were removed. The remaining studies underwent a three-step screening process. First, titles were screened to identify potentially relevant studies. Abstracts were then screened to ensure that they met the inclusion criteria. Finally, full-text screening was conducted on the remaining studies to confirm their eligibility for inclusion in the review. For each included study, relevant data were extracted, including details about the Sudoku task design, neuroimaging techniques used and key neural findings. The studies were categorized based on the neuroimaging method employed, allowing for a structured analysis of the neural correlates of chess expertise across different modalities. This process is demonstrated in Figure 2, included studies are summarised in Table 1.

**Table 1:** Studies included in the systematic review (*N* = 6).

Author/s	Year	Neuroimaging modality	Imaging parameters	Sample size + Mean age (MA)	Population	Difficulty manipulation	Key findings	Coordinates identified	Name of paper	DOI
Ashlesh	2020	fNIRS	Optode configuration: 4 sources, 10 detectors 16 measurement channels (optodes) Positioned over prefrontal cortex (Fp1, Fp2, Fz reference using 10–20 system) Forehead montage only (no whole-brain coverage) Wavelengths: 730 nm 850 nm Baseline recording: 10 s prescan baseline Filtering & preprocessing: Low-pass filter: 0.40 Hz (order 20) Wavelet minimum description length detrending Precoloring HRF filter for temporal autocorrelation correction Hemodynamic measures: Oxyhemoglobin (HbO) Deoxyhemoglobin (HbR) Modified Beer–Lambert Law	33 (*F* = 9) MA = 28.88	Healthy	Step 1 (Lower difficulty) 3 × 3 isolated grids Only subgrid rule Step 2 (Higher difficulty) Full 9 × 9 matrix	Both the medial and lateral regions of PFC are activated when the participant is solving the Sudoku task	Not applicable for neuroimaging modality utilised	Role of prefrontal cortex during Sudoku task: fNIRS study	10.1515/tnsci-2020-0147
Jin	2012	fMRI	Structural Imaging: TR = 500 ms TE = 8.5 ms FOV = 220 × 220 mm Matrix = 256 × 256 Slice thickness = 3.4 mm Slice gap = 0 mm 35 continuous axial slices Alignment: AC–PC line Functional Imaging: Sequence: Single-shot echo-planar imaging (EPI) TR = 2000 ms TE = 25 ms Flip angle = 79° FOV = 220 × 220 mm Matrix = 64 × 64 Slice thickness = 3.4 mm Slice gap = 0 mm 35 slices Run duration ≈ 12 min Two sessions per participant	18 (*F* = 7) MA = 63.6	Healthy	Simple Task Solve using row rule or column rule Only one heuristic required. Complex Task Solve using both row and column rules	On both tasks, the participants showed deactivation in the bilateral posterior cingulate cortex (PCC)/precuneus regions. The extent of deactivation on the complex task was greater than that on the simple task.	Left PCC/Precuneus center: x = −7, y = −48, z = 27 Right PCC/Precuneus center: x = −5, y = −48, z = 27	fMRI study in posterior cingulate and adjacent precuneus cortex in healthy elderly adults using problem solving task	10.1016/j.jns.2012.02.032
Nombela	2011	fMRI	Functional Imaging Parameters: Sequence: Echo-planar imaging (EPI) TR = 3,000 ms TE = 50 ms Flip angle = 90° FOV = 230 × 230 mm Matrix = 128 × 128 28 contiguous slices Slice thickness = 3.0 mm Slice gap = 1 mm In-plane resolution ≈ 2.5 × 1.8 mm Voxel size after normalization = 3 × 3 × 3 mm Functional run duration = 9 min 6 s Structural Imaging: High-resolution 3D anatomical acquisition T1-weighted images matched to functional slices	10 (*F* = 6) MA = 60.5	Parkinsons	Sudoku difficulty was not manipulated	At the first evaluation, trained PD patients did not differ significantly in brain activation compared to untrained PD patients during the Stroop task. At the second evaluation, trained PD patients showed greater activation than untrained PD patients in: Right superior and medial temporal gyri, suggesting potential training-related effects in these areas. Compared to controls, trained PD patients at the second evaluation showed greater activation in: Right anterior cingulate gyrus Left inferior frontal gyrus Right medial frontal gyrus Left angular and precuneus gyri Right supramarginal gyrus Left superior parietal gyrus Right medial temporal gyrus Left parahippocampal gyrus Left insula and thalamus	First Evaluation – Incongruent > Congruent (PD Patients) Right Superior Frontal Gyrus: x = 6, y = 9, z = 51 Left Superior Frontal Gyrus: x = −6, y = 15, z = 48 Left Precentral Gyrus: x = −36, y = −6, z = 30 Right Supramarginal Gyrus: x = 42, y = −51, z = 33 Right Inferior Parietal Gyrus: x = 42, y = −30, z = 39 Left Superior Temporal Gyrus: x = −54, y = −57, z = 27 Right Anterior Cingulate Gyrus: x = 18, y = −6, z = 27 Right Putamen: x = 24, y = −3, z = −3 Left Caudate Tail: x = −18, y = −27, z = 18 Left Parahippocampal Gyrus: x = −27, y = −24, z = −9 Left Lingual Gyrus: x = −30, y = −75, z = −6 Control > PD (First Evaluation) Left Precentral Gyrus: x = −36, y = −12, z = 36 Left Medial Frontal Gyrus: x = −6, y = 39, z = 42 Right Precuneus Gyrus: x = 3, y = −57, z = 45 Left Inferior Parietal Gyrus: x = −45, y = −24, z = 27 Second Evaluation – Untrained > Trained PD Right Precentral Gyrus: x = 39, y = 6, z = 36 Left Superior Frontal Gyrus: x = −27, y = 36, z = 30 Right Medial Frontal Gyrus: x = 6, y = 51, z = −9 Left Medial Frontal Gyrus: x = −42, y = 44, z = 24 Left Primary Somatosensory Cortex: x = −48, y = −21, z = 33 Left Precuneus Gyrus: x = −12, y = −54, z = 36 Left Angular Gyrus: x = −36, y = −69, z = 30 Left Superior Temporal Gyrus: x = −45, y = −21, z = 3 Left Superior Temporal Gyrus (posterior): x = −63, y = −36, z = 6 Right Cuneus Gyrus: x = 6, y = −78, z = 21 Left Cuneus Gyrus: x = −9, y = −78, z = 21 Left Lingual Gyrus: x = −9, y = −66, z = 0 Left Amygdala: x = −9, y = −57, z = −36 Left Anterior Cingulate Gyrus: x = −9, y = 21, z = −3 Right Posterior Cingulate Gyrus: x = 18, y = −60, z = 12 Right Putamen: x = −24, y = −15, z = 6 Left Ventrolateral Thalamus: x = −15, y = −12, z = 9 Left Anterior Culmen (Cerebellum): x = 0, y = −60, z = 3 Left Uvula (Cerebellum): x = −3, y = −66, z = −30 Right Anterior Lobe (Cerebellum): x = 15, y = −51, z = −3	Cognitive Rehabilitation in Parkinson’s Disease: Evidence from Neuroimaging	10.3389/fneur.2011.00082
Qin	2012	fMRI	Functional Imaging Parameters: Sequence: Gradient echo-planar imaging (EPI) TR = 2000 ms TE = 31 ms Flip angle = 90° Matrix = 64 × 64 FOV = 200 × 200 mm 32 axial slices Slice thickness = 3.2 mm Slice gap = 0 mm Voxel size = 3.125 × 3.125 × 3.2 m AC–PC aligned (23rd slice reference) 14 s dummy scans before acquisition	15 (*F* = 8) MA = 23.1	Healthy	Sudoku difficulty was not manipulated	All five ROIs (left fusiform gyrus, prefrontal cortex, posterior parietal cortex, caudate, and dorsal ACC) showed task-positive BOLD responses.	Peak Talairach coordinates were not reported in the provided excerpt. ROIs were defined either *a priori* or identified via exploratory whole-brain analysis. ACT-R Predefined ROIs (Talairach Coordinates) Right Fusiform Gyrus (BA 37): x = 42, y = −60, z = −8 Left Fusiform Gyrus (BA 37): x = −42, y = −60, z = −8 Right Caudate Nucleus: x = 15, y = 9, z = 2 Left Caudate Nucleus: x = −15, y = 9, z = 2 Right Prefrontal Cortex (BA 45/46): x = 40, y = 21, z = 21 Left Prefrontal Cortex (BA 45/46): x = −40, y = 21, z = 21 Right Posterior Parietal Cortex (BA 7/39/40): x = 23, y = −64, z = 31 Left Posterior Parietal Cortex (BA 7/39/40): x = −23, y = −64, z = 31 Right Anterior Cingulate Cortex (BA 24/32): x = 5, y = 10, z = 38 Left Anterior Cingulate Cortex (BA 24/32): x = −5, y = 10, z = 38	Neural bases for basic processes in heuristic problem solving: Take solving Sudoku puzzles as an example	10.1002/pchj.15
Qiu	2018	fMRI	Imaging Parameters: Sequence: Single-shot gradient-echo T2 EPI TR = 2000 ms TE = 30 m Flip angle = 90° Slice thickness = 3.0 mm In-plane resolution = 3.0 × 3.0 mm Field of view = 192 × 192 mm 38 axial slices Interleaved acquisition Slices parallel to AC–PC line	21 (*F* = 12)	Healthy	Difficulty Manipulation: 10 difficulty levels Defined by minimum number of logic steps Staircase procedure: Increased after two correct trials Decreased after two incorrect trials	In the Sudoku task, metacognitive processing during redecision primarily involved the lateral frontopolar cortex (lFPC), which was responsible for metacognitive control—adjusting decisions based on uncertainty. The dorsal anterior cingulate cortex (dACC) and anterior insular cortex (AIC) supported metacognitive monitoring, tracking how uncertain a decision felt. Additionally, the ventral striatum (VS) was uniquely active in Sudoku and appeared to motivate control, with its interaction with the lFPC predicting how much individuals improved their accuracy. Other regions like the middle DLPFC and aIPL were part of the broader control network but had less clearly defined roles.	Coordinates not reported in original study.	The neural system of metacognition accompanying decision-making in the prefrontal cortex	10.1371/journalpbio.2004037
Su	2022	fMRI	Imaging Parameters: Sequence: Gradient-echo EPI (T2*) TR = 2000 ms TE = 30 ms Flip angle = 90° Slice thickness = 3.0 mm In-plane resolution = 3.0 × 3.0 mm Field of view = 192 × 192 mm 38 axial slices Interleaved acquisition Slices aligned parallel to AC–PC line	22 (M = 12) MA = 24.5	Healthy	Difficulty defined by minimum number of logic steps Staircase procedure during scanning: Difficulty increased after 2 correct + high confidence Difficulty decreased after 2 incorrect + low confidence	Dorsal anterior cingulate cortex (dACC) activity increased during the second decision phase when participants were re-evaluating their initial answer, reflecting metacognitive control. Precuneus, DLPFC and interparietal sulcus also involved.	Coordinates not reported in original study.	Task-Specific Neural Representations of Generalizable Metacognitive Control Signals in the Human Dorsal Anterior Cingulate Cortex	10.1523/JNEUROSCI.1283-21.2021

## 3 Results

A total of six neuroimaging studies met the inclusion criteria and were included in the review. These publications span from 2011 to 2025. Across studies, the majority were conducted in healthy adult participants, while one study included patients with Parkinson’s disease (Nombela et al., 2011). Where reported, participant characteristics varied, with most samples comprising healthy adults (Table 2).

**Table 2:** Experimental task design and comparison conditions.

Study	Task description	Response method	Experimental design	Comparison condition/contrast	Purpose of contrast
Ashlesh et al. (2020)	Participants solved Sudoku puzzles of varying grid sizes during fMRI scanning	Button press	Block design	Sudoku blocks vs. rest	To identify neural activity associated with puzzle solving
Jin et al. (2012)	Participants completed rule-based Sudoku puzzles requiring logical inference	Button press	Block design	Complex vs. simple Sudoku conditions	To identify regions involved in rule-based reasoning
Nombela et al. (2011)	Participants completed a cognitive training program including Sudoku tasks	Various responses	Pre–post design	Pre-training vs. post-training Stroop task	To assess training-related changes in cognitive control networks
Qin et al. (2012)	Participants solved 4 × 4 Sudoku puzzles with varying rule complexity	Button press	Event-related design	Complex rule vs. simple rule; one-step vs. two-step inference	To isolate neural activity associated with reasoning complexity
Qiu et al. (2018)	Participants completed Sudoku puzzles with varying difficulty levels	Button press	Event-related design	High difficulty vs. low difficulty	To examine neural correlates of task difficulty
Su et al. (2022)	Participants performed decision and re-decision phases while solving Sudoku puzzles	Button press	Event-related design	Initial decision vs. re-decision	To investigate neural mechanisms of decision evaluation

Five studies used functional magnetic resonance imaging (fMRI), a technique that measures blood-oxygen-level-dependent (BOLD) responses as a proxy for neuronal activity. Nombela et al. (2011) differed from the other included studies in that it employed a longitudinal Sudoku-based cognitive training intervention in patients with Parkinson’s disease. Neuroimaging was conducted during a Stroop task rather than during Sudoku performance itself. As such, this study provides indirect evidence of training-related neural changes rather than direct insight into the neural correlates of Sudoku solving.

In healthy young adults, Qin et al. (2012) asked participants to solve 4 × 4 Sudoku puzzles in the fMRI scanner, focusing on regions of interest including the prefrontal cortex, posterior parietal cortex, fusiform gyrus, caudate, and dorsal anterior cingulate cortex (ACC), thereby linking Sudoku to heuristic problem solving. Jin et al. (2012) studied elderly participants performing simple and complex 4 × 4 tasks, demonstrating increased deactivation of default mode network regions, particularly the posterior cingulate cortex and precuneus, during more complex puzzles. Two later studies examined metacognitive processes. Qiu et al. (2018) used a re-decision paradigm, implicating the lateral frontopolar cortex, anterior cingulate, and striatal regions in monitoring and control. Similarly, Su et al. (2022) employed a two-phase decision task under uncertainty, highlighting dorsal ACC involvement alongside dorsolateral prefrontal and intraparietal regions.

Beyond fMRI, one study broadened the methodological scope. Ashlesh et al. (2020) applied functional near-infrared spectroscopy (fNIRS), a haemodynamic technique that enables more ecologically valid experimental setups. Participants completed paper-and-pencil Sudoku tasks progressing from simplified subgrids to full 9 × 9 puzzles. Analyses of oxygenated and deoxygenated haemoglobin revealed increased prefrontal activation with increasing task complexity.

Across the included studies, Sudoku tasks were implemented using either event-related or block designs in which participants solved simplified puzzles while undergoing neuroimaging. Participants typically responded using button presses to indicate the correct number for a target cell or to confirm completion of a puzzle (Table 2). Tasks were contrasted against baseline or control conditions such as rest, fixation, or simpler rule-based conditions to isolate neural activity associated with problem solving.

### 3.1 Evidence summary: task characteristics

Across the included studies, Sudoku tasks ranged from simplified 4 × 4 puzzles to standard 9 × 9 grids with 3 × 3 sub grids, reflecting differences in task difficulty and potentially cognitive load. One study (Ashlesh et al., 2020) implemented the traditional 9 × 9 grid format, whilst five studies implemented a 4 × 4 grid design (Jin et al., 2012; Nombela et al., 2011; Qin et al., 2012; Qiu et al., 2018; Su et al., 2022). These smaller grids were chosen to facilitate task completion within the constraints of neuroimaging sessions and to enable controlled manipulation of task difficulty.

Task complexity was manipulated in multiple ways. Jin et al. (2012) explicitly contrasted simple versus complex 4 × 4 puzzles, showing that higher difficulty levels modulated activation in default mode regions. Ashlesh et al. (2020) similarly structured their design to escalate from a subgrid-only condition to a full-rule Sudoku, observing greater medial prefrontal activation under the more demanding condition. Qiu et al. (2018) and Su et al. (2022) both employed a decision re-decision paradigm, which required participants to make an initial judgment and subsequently revisit their decision, a design that taxed both logical reasoning and metacognitive control. Qin et al. (2012) explored Sudoku as a heuristic problem-solving task, emphasizing the role of rule application and flexibility rather than direct manipulations of puzzle complexity. Together, these studies illustrate that Sudoku lends itself to diverse methodological approaches for systematically increasing cognitive load.

Although individual cognitive phases were not explicitly isolated, several studies manipulated puzzle difficulty or decision structure, providing indirect insight into demand-dependent neural recruitment during Sudoku solving.

### 3.2 Evidence summary: neuroimaging modalities implemented

The six included studies employed 2 distinct neuroimaging modalities, fMRI and fNIRS. Most investigations (Nombela et al., 2011; Jin et al., 2012; Qin et al., 2012; Qiu et al., 2018; Su et al., 2022) used fMRI to capture blood-oxygen-level-dependent (BOLD) responses during Sudoku solving. Ashlesh et al. (2020) provided the only fNIRS study (consisting of a 16-channel forehead sensor array), measuring hemodynamic changes in the prefrontal cortex during paper-and-pencil Sudoku. Taken together, the use of multiple neuroimaging modalities underscores the multidimensional nature of Sudoku-related brain activity.

### 3.3 Evidence summary: key neural findings

The neuroimaging literature on Sudoku solving reveals consistent engagement of frontoparietal control networks associated with working memory and executive functions, alongside task-dependent variations.

Across studies, the prefrontal cortex emerged as a central hub for Sudoku-related activity. Ashlesh et al. (2020) demonstrated increased oxygenated haemoglobin in medial and lateral prefrontal regions, with activation scaling alongside task complexity. fMRI studies provided more detailed localisation. Qin et al. (2012) reported robust prefrontal activation within a broader problem-solving network including the posterior parietal cortex, fusiform gyrus, caudate, and dorsal ACC. Qiu et al. (2018) and Su et al. (2022) further identified the lateral frontopolar cortex as critical for metacognitive control, particularly during decision revision under uncertainty, alongside involvement of the dorsolateral prefrontal cortex.

The dorsal anterior cingulate cortex was consistently implicated in monitoring and control processes. Increased activation was observed during re-decision phases, supporting its role in detecting conflict and guiding behavioural adjustment. Nombela et al. (2011) similarly reported increased ACC activation following Sudoku-based training, although this was measured during a Stroop task and therefore reflects indirect evidence of neural change.

Parietal regions, including the inferior parietal lobule and intraparietal sulcus, were also consistently engaged, reflecting their roles in visuospatial processing, numerical cognition, and working memory. In contrast, Jin et al. (2012) demonstrated deactivation of default mode network regions, including the posterior cingulate cortex and precuneus, with greater suppression observed during more complex tasks. This suggests that Sudoku solving involves both activation of executive control networks and suppression of internally directed processes under increased cognitive demand.

Finally, Qiu et al. (2018) identified ventral striatal activity associated with reductions in decision uncertainty, with functional coupling between the ventral striatum and frontopolar cortex predicting improved performance during re-decision.

## 4 Discussion

### 4.1 Overview of neural systems engaged during Sudoku solving

**Figure 3:**
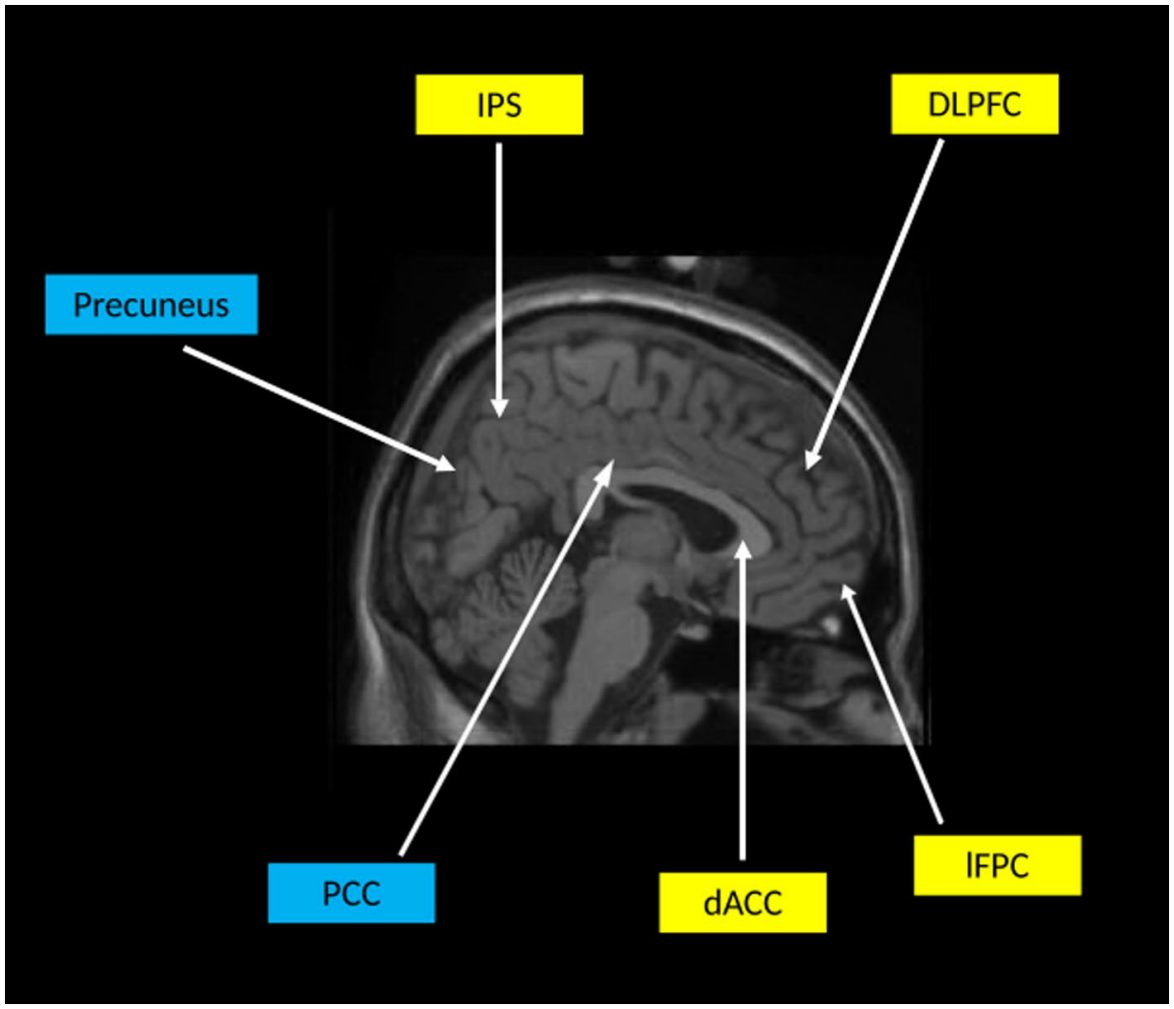
Conceptual schematic of neural systems engaged during Sudoku solving. Regions highlighted in yellow represent components of the frontoparietal control network consistently activated across the literature, including the dorsolateral prefrontal cortex and intraparietal sulcus. Reflecting executive control and working memory demands. Regions highlighted in yellow also include monitoring and metacognitive control areas, such as the dorsal anterior cingulate cortex and lateral frontopolar cortex, which are engaged during error monitoring and uncertainty resolution. Regions highlighted in blue represent default mode network areas, including the posterior cingulate cortex and precuneus, which show task-related deactivation during Sudoku solving. This schematic emphasises convergence at the level of large-scale neural systems rather than precise anatomical localisation.

This systematic review identified six neuroimaging studies that have examined neural activity during Sudoku related tasks. Five publications employed functional MRI, with additional evidence provided by one fNIRS study. Across studies, overlapping activation was reported. Across studies, Sudoku engaged frontoparietal executive control networks, with core regions including the DLPFC, dACC, IPS, lFPC and AIC. These areas collectively support cognitive control, monitoring of uncertainty and adjustment of problem-solving strategies. Beyond executive control, studies also highlight motivational influences, with ventral striatal activation consistent with involvement of reward-related circuitry. Suppression of default mode network activity, particularly within the posterior cingulate and precuneus, further indicates a shift away from internally oriented processes during complex problem solving. These core neural systems and their functional relationships are summarised schematically in Figure 3.

### 4.2 Executive control, working memory, and metacognitive monitoring

The findings of this review suggest that Sudoku engages a broad range of neural systems underpinning executive control and metacognitive monitoring. A consistent finding is the involvement of the DLPFC and IPS during Sudoku solving (Qin et al., 2012; Qiu et al., 2018; Su et al., 2022). Both regions are core components of the frontoparietal control network and are well established in the broader literature as supporting WM maintenance and attentional allocation (Dixon et al., 2018; Duncan, 2010; Harding et al., 2015; Murray et al., 2017; Spreng et al., 2010). The recruitment of these neural areas during Sudoku playing is consistent with task demands requiring simultaneously tracking multiple constraints and dynamically updating potential solutions. Furthermore, the dACC and lFPC emerge as critical substrates for metacognitive monitoring and control (Qiu et al., 2018; Su et al., 2022). The dACC is widely implicated in conflict detection and error monitoring (Bush et al., 2002; Heilbronner and Hayden, 2016; Shenhav et al., 2016), while the lFPC has been linked to higher-order re-evaluation processes, including the weighing of uncertainty and strategy (Boorman et al., 2011; Mansouri et al., 2017: Pollmann, 2001). Sudoku paradigms that incorporate multiple evaluation phases have demonstrated that activity in these regions scales with subjective uncertainty, consistent with their proposed role in second-order cognitive monitoring. Importantly, Sudoku also engages motivational circuitry. The results from Qiu et al. (2018) demonstrated that VS activity tracked reductions in decision uncertainty and predicted accuracy improvements, consistent with involvement of reward-related processes during uncertainty reduction. This aligns with a broader literature on the VS in encoding the motivational value of cognitive effort and error reduction (Groenewegen et al., 1999; Heimer et al., 1982; Kelley, 2004). Taken together, Sudoku tasks elicit a distributed but convergent pattern of neural activity encompassing executive control, error monitoring and motivational systems.

### 4.3 Sudoku task phases, difficulty, and demand-dependent neural engagement

Sudoku solving is not a monolithic cognitive operation but instead comprises multiple components, including visual scanning, candidate elimination, rule application, and multi-step deductive reasoning, with cognitive demands increasing as puzzle difficulty rises. Importantly, however, most neuroimaging studies included in this review did not explicitly segment these phases, instead examining overall task engagement or manipulating puzzle difficulty and decision structure as proxies for cognitive demand.

Despite this limitation, convergent patterns across studies suggest graded, demand-dependent neural recruitment rather than discrete neural signatures for individual Sudoku phases. Simpler or early-stage problem solving, which may place greater emphasis on visual search and visuospatial working memory, was associated with engagement of posterior parietal regions and visual processing areas (Qin et al., 2012). In contrast, higher task demands, operationalised through increased puzzle complexity (Jin et al., 2012; Ashlesh et al., 2020) or through re-decision paradigms requiring re-evaluation under uncertainty (Qiu et al., 2018; Su et al., 2022) consistently recruited prefrontal regions including the DLPFC, dACC, and lateral frontopolar cortex, reflecting increased executive control, conflict monitoring, and metacognitive processing. Taken together, the available evidence indicates that Sudoku engages a common frontoparietal control architecture whose intensity and configuration vary with cognitive demand, rather than revealing sharply dissociable neural signatures for distinct task phases. Future studies employing time-resolved analyses or explicit phase segmentation will be required to delineate the neural dynamics associated with individual components of Sudoku solving.

### 4.4 Multimodal integration and methodological considerations

The interpretation of neural correlates of Sudoku solving is complicated by the use of multiple neuroimaging modalities across studies, each characterised by distinct spatial and temporal sensitivities. Functional MRI and fNIRS index haemodynamic responses that provide relatively high spatial resolution but reflect neural activity indirectly and on the order of seconds. These differences constrain the extent to which findings can be directly compared or integrated across modalities. In the present literature, haemodynamic methods have primarily identified the cortical loci engaged during Sudoku solving, consistently implicating frontoparietal and cingulo-opercular regions associated with executive control and monitoring. Importantly, these oscillatory findings do not map straightforwardly onto specific anatomical regions identified by fMRI or fNIRS, but instead reflect distributed network-level processes. As a result, apparent discrepancies across modalities should not be interpreted as conflicting evidence, but rather as complementary perspectives on a shared underlying cognitive architecture. Haemodynamic activation patterns describe where control processes are engaged, while electrophysiological measures capture how rapidly and dynamically these processes unfold. However, the limited number of studies and lack of multimodal recordings within the same participants currently preclude direct cross-modal validation. Future work combining fMRI, EEG, and fNIRS within unified experimental designs will be essential to fully characterise the spatiotemporal dynamics of Sudoku-related cognition.

### 4.5 Sudoku in relation to other cognitive training paradigms

A central finding across the results is the consistent recruitment of the frontoparietal network, particularly regions implicated in WM and attentional control. The DLPFC and IPS are key neural regions in the WM system, as they are involved in the maintenance, updating and manipulation of information under conditions significant cognitive load (Barbey et al., 2013; Curtis and D’Esposito, 2003; Bray et al., 2015; Offen et al., 2010; Webler et al., 2022). These results align Sudoku with established WM paradigms including the n-back task, the Tower of London and mental arithmetic tasks. Each of these tasks elicit DLPFC-parietal co-activation and has been widely used to operationalise executive function (Chang et al., 2011; DeStefano and LeFevre, 2004; Gajewski et al., 2018; Harvey et al., 2005; Nitschke et al., 2017; Takeuchi et al., 2011; Van Impe et al., 2011).

To situate Sudoku within the broader cognitive training literature, it is useful to compare it directly with commonly used paradigms such as the n-back task and mental arithmetic. These tasks are frequently employed to probe and train working memory and executive control due to their well-defined cognitive structure and robust engagement of frontoparietal networks. The n-back task isolates continuous updating and maintenance of information under time pressure, typically engaging the dorsolateral prefrontal cortex and intraparietal sulcus in a highly constrained and repetitive manner (Harvey et al., 2005). Mental arithmetic similarly recruits frontoparietal and cingulate regions (Fehr et al., 2007; Menon et al., 2000) but places greater emphasis on numerical manipulation and speeded processing. In contrast, Sudoku integrates multiple executive processes within a single task, including visuospatial working memory, rule maintenance, hypothesis testing, conflict monitoring, and metacognitive evaluation. This composite structure more closely resembles real-world problem solving, where cognitive demands shift dynamically rather than remaining fixed. From a translational perspective, Sudoku differs from traditional laboratory paradigms in its ecological validity and accessibility. Whereas tasks such as the n-back or mental arithmetic are typically implemented in controlled experimental settings, Sudoku is widely available outside laboratory environments and is commonly performed in a self-paced format. Motivation is a critical determinant of adherence in cognitive training interventions, and tasks that are intrinsically rewarding may better sustain long-term engagement. These characteristics indicate potential feasibility for longitudinal interventions, although empirical validation is required.

### 4.6 Translational potential

Beyond its value as an experimental task, the results from the present review display Sudoku as a potential candidate for cognitive training. The consistent involvement of neural regions such as DLPFC, dACC and lFPC suggests that potentially engaging in Sudoku could strengthen the neural circuits underlying WM and cognitive control (Duda and Sweet, 2020; Morrison and Chein, 2011; Shipstead et al., 2012). Also, Sudoku tasks drive suppression of the PCC and precuneus within the default mode network, a pattern frequently associated with task engagement and externally directed cognition (Jin et al., 2012). These converging findings point to Sudoku as a robust probe of executive function, with implications for translational application. Early evidence supports this possibility: in Parkinson’s disease, a six-month Sudoku-based training programme was associated with enhanced fronto-temporal and parietal activation, interpreted as a marker of functional reorganisation and neuroplasticity (Nombela et al., 2011).

Similar approaches may offer a low-cost, accessible means of maintaining executive function in older adults. From a methodological perspective, portable neuroimaging techniques (fNIRS) extend Sudoku’s translational viability to bedside, home-based, or rehabilitation contexts. Ashlesh et al. (2020) demonstrated that fNIRS captures reliable haemodynamic changes in prefrontal cortex during Sudoku solving. These modalities are lightweight, non-invasive, and increasingly affordable, making them well-suited for longitudinal monitoring in clinical and real-world environments compared to static, more expensive fMRI. A further translational advantage of Sudoku as a form of cognitive training lies in its ecological validity and widespread availability. Unlike traditional cognitive training paradigms, such as the n-back task or arithmetic drills, Sudoku is widely familiar and commonly performed in non-clinical contexts. Motivation has been highlighted as a critical factor in sustaining engagement with cognitive training interventions (Katz et al., 2014; Mohammed et al., 2017). Sudoku is commonly undertaken voluntarily in non-clinical contexts, which could influence adherence, although this has not been empirically tested. This motivational dimension may be particularly relevant in older adults, where compliance often limits the effectiveness of cognitive training protocols. Digital platforms may amplify these opportunities. Online forms of Sudoku allow for dynamic control of task difficulty, progression that is personalised and adaptive features that maintain performance within an optimal challenge range.

Nevertheless, an important question is whether Sudoku-based training can produce benefits that generalise beyond the puzzle domain itself. While the evidence from this systematic review suggest Sudoku robustly engages frontoparietal and metacognitive networks, more evidence is required to determine whether these neural correlates can translate into improvements into daily functioning and problem-solving, and also compared to other forms of cognitive training.

A comparison with the literature on the neural correlates of chess playing as a potential form of cognitive training intervention, highlights both overlaps and distinctions in cognitive demands when compared to Sudoku playing. Both Sudoku and chess recruit the frontoparietal network, particularly the DLPFC and IPS, consistent with their reliance on working memory, attentional control, and multi-step reasoning (Leong et al., 2024; Qin et al., 2012; Qiu et al., 2018; Williams et al., 2025). Chess, however, places stronger demands on visual–perceptual expertise, reflected in consistent engagement of occipito-temporal regions including the fusiform gyrus and in altered connectivity in thalamic and parietal circuits among expert players (Williams et al., 2025). Translationally, this suggests that chess may be better suited for interventions aimed at visuospatial expertise and strategic planning, whereas Sudoku provides a more flexible, rule-based paradigm for probing and potentially training working memory, cognitive control, and metacognitive processes. In addition, while chess has also been proposed as a cognitive training paradigm, demographic differences in participation have been reported, with some studies indicating higher participation rates among males (Williams et al., 2025). Conversely, Sudoku is more modern and potentially more accessible to all genders popularised by Japanese Maki Kaji in the 1980s and is typically played individually without the added pressure of competing. Both chess and Sudoku training however, require rigorous longitudinal studies to establish whether neural engagement translates into generalisable cognitive benefits.

### 4.7 Limitations of the current evidence base

Several limitations should be acknowledged when interpreting the findings of this systematic review. The number of available neuroimaging studies of Sudoku remains very small, with only six eligible publications identified, highlighting the need for more neuroimaging studies of Sudoku. This restricted evidence base limits the strength of any conclusions and increases the likelihood that individual study biases, such as small sample sizes or methodological choices, disproportionately shape the observed patterns. Furthermore, most studies relied on cross-sectional designs and single-session imaging, limiting the ability to assess training effects or causal relationships between Sudoku engagement and neural recruitment. Only one study investigated longitudinal outcomes following extended Sudoku training, and even this was in a highly specific clinical context concerning people with Parkinson’s disease (Nombela et al., 2011). Finally, methodological differences across imaging modalities introduce additional complexity. While fMRI dominated the literature, one study employed fNIRS and zero employed EEG. These techniques differ in spatial and temporal resolution, sensitivity to cortical versus subcortical structures, and susceptibility to artefacts, making integration across modalities less straightforward. Taken together, these limitations highlight the need for caution when extrapolating the current findings and underscore the importance of future research employing larger, more diverse samples, harmonised task protocols, and longitudinal designs.

### 4.8 Future directions

The current body of Sudoku neuroimaging research remains limited in scope. Future studies will need to address several key gaps to establish Sudoku as both a robust experimental paradigm and a viable candidate for cognitive training. Larger and more diverse sample sizes are essential. Most existing studies involve relatively small groups of healthy young adults, with only one study in a clinical population (Nombela et al., 2011). Expanding research to include people with psychological disorders would provide critical evidence on the generalisability of Sudoku-based interventions. Furthermore, systematic comparisons of task variations are warranted. Differences in grid size (3 × 4; 4 × 4; 9 × 9), complexity levels and decision phases (initial decision vs. re-evaluation) modulate neural recruitment (Jin et al., 2012; Qiu et al., 2018; Su et al., 2022). Directly contrasting these task variants within the same experimental design would clarify how cognitive load, rule complexity, and metacognitive demands shape frontoparietal and motivational network engagement.

Integration with clinical trials represents an important translational step. Given its accessibility and adaptability, Sudoku could easily be implemented within cognitive enhancement programmes. Trials and evidence in populations with conditions with a risk of executive dysfunction could evaluate changes in neural activity as well as real-world outcomes such as daily functioning and quality of life. If Sudoku-based training proves effective in these settings, it could provide a low-cost, engaging, and evidence-based intervention to support cognitive health across the lifespan.

## 5 Conclusion

This systematic review synthesised a small group of studies that have examined the neural correlates of Sudoku playing. Across six studies employing fMRI and fNIRS Sudoku consistently engaged frontoparietal executive control networks (notably DLPFC and IPS), alongside regions supporting metacognitive monitoring (dACC, lFPC) and motivational drive (ventral striatum). While the literature remains limited in scope and heterogeneous in design, these findings indicate that Sudoku is a valuable probe of WM, cognitive control and metacognitive processes, with promising translational potential as an engaging, accessible cognitive training paradigm. Further research will need to address limitations by employing larger, more diverse samples, harmonised task designs and longitudinal multimodal imaging approaches, as well as embedding Sudoku-based training within clinical trials. In doing so, it may be possible to establish Sudoku not only as a window into the neural architecture of executive function but also as a practical tool for supporting cognitive health.

## Data Availability

Publicly available datasets were analyzed in this study. This data can be found here: LJMU stored available on request.
